# Application of calibrated fMRI in Alzheimer's disease

**DOI:** 10.1016/j.nicl.2017.05.009

**Published:** 2017-05-16

**Authors:** Isabelle Lajoie, Scott Nugent, Clément Debacker, Kenneth Dyson, Felipe B. Tancredi, AmanPreet Badhwar, Sylvie Belleville, Yan Deschaintre, Pierre Bellec, Julien Doyon, Christian Bocti, Serge Gauthier, Douglas Arnold, Marie-Jeanne Kergoat, Howard Chertkow, Oury Monchi, Richard D. Hoge

**Affiliations:** a Département de Pharmacologie et physiologie, Université de Montréal, Montreal, QC, Canada; b Centre de Recherche de l'Institut Universitaire de Gériatrie de Montréal, Montreal, QC, Canada; c Montreal Neurological Institute, Montreal, QC, Canada; d Department of Neurology and Neurosurgery, McGill University, Montreal, QC, Canada; e Department of Psychiatry, McGill University, Montreal, QC, Canada; f Department of Physiology, McGill University, Montreal, QC, Canada; g Departamento de Radiologia, Centro de Pesquisa em Imagem, Hospital Israelita Albert Einstein, São Palo, SP, Brazil; h Department of Computer Science and Operations Research, Université de Montréal, Montreal, QC, Canada; i Département de Psychologie, Université de Montréal, Montreal, QC, Canada; j Département de Neurosciences, Université de Montréal, Montreal, QC, Canada; k Service de neurologie, Département de Médecine, CHUM Notre-Dame, Montréal, QC, Canada; l Division de Neurologie, Faculté de Médecine et des Sciences de la Santé & Research Centre on Aging, Université de Sherbrooke, Sherbrooke, QC, Canada; m McGill Center for Studies in Aging, Douglas Mental Health Research Institute, Montreal, QC, Canada; n Département de Médecine, Université de Montréal, Montreal, QC, Canada; o Department of Medicine, Sir Mortimer B. Davis-Jewish General Hospital, McGill University, Montreal, QC, Canada; p Department of Clinical Neurosciences and Hotchkiss Brain Institute, University of Calgary, Calgary, AB, Canada

**Keywords:** Calibrated fMRI, Alzheimer's disease, Cerebral blood flow, Oxidative metabolism, Oxygen extraction fraction, BOLD calibration constant, Cerebrovascular reactivity

## Abstract

Calibrated fMRI based on arterial spin-labeling (ASL) and blood oxygen-dependent contrast (BOLD), combined with periods of hypercapnia and hyperoxia, can provide information on cerebrovascular reactivity (CVR), resting blood flow (CBF), oxygen extraction fraction (OEF), and resting oxidative metabolism (CMRO_2_). Vascular and metabolic integrity are believed to be affected in Alzheimer's disease (AD), thus, the use of calibrated fMRI in AD may help understand the disease and monitor therapeutic responses in future clinical trials. In the present work, we applied a calibrated fMRI approach referred to as Quantitative O2 (QUO2) in a cohort of probable AD dementia and age-matched control participants. The resulting CBF, OEF and CMRO_2_ values fell within the range from previous studies using positron emission tomography (PET) with ^15^O labeling. Moreover, the typical parietotemporal pattern of hypoperfusion and hypometabolism in AD was observed, especially in the precuneus, a particularly vulnerable region. We detected no deficit in frontal CBF, nor in whole grey matter CVR, which supports the hypothesis that the effects observed were associated specifically with AD rather than generalized vascular disease. Some key pitfalls affecting both ASL and BOLD methods were encountered, such as prolonged arterial transit times (particularly in the occipital lobe), the presence of susceptibility artifacts obscuring medial temporal regions, and the challenges associated with the hypercapnic manipulation in AD patients and elderly participants. The present results are encouraging and demonstrate the promise of calibrated fMRI measurements as potential biomarkers in AD. Although CMRO_2_ can be imaged with ^15^O PET, the QUO2 method uses more widely available imaging infrastructure, avoids exposure to ionizing radiation, and integrates with other MRI-based measures of brain structure and function.

## Highlights

• An advanced calibrated fMRI method is employed to explore brain physiology in AD.
• A pattern of hypoperfusion and hypometabolism was observed as in PET studies.
• The method provides several potential biomarkers of interest in AD.
• The technique may help by providing new insights into the pathophysiology of AD.

## 1 Introduction

Functional MRI (fMRI) methods, such as brain imaging based on arterial spin-labeling (ASL) or blood oxygenation level-dependent (BOLD) contrasts, are sensitive to cerebral blood flow (CBF) and the cerebral metabolic rate of oxygen (CMRO_2_). Calibrated fMRI exploits this sensitivity, using ASL and BOLD data acquired during controlled manipulations of cerebral physiology to compute quantitative estimates of CMRO_2_ and related parameters such as the BOLD calibration constant *M* (Davis et al., 1998, Chiarelli et al., 2007, Gauthier and Hoge, 2012).

In contemporary calibrated fMRI methods, which combine hypercapnic and hyperoxic manipulations, absolute measures of resting CMRO_2_ in addition to other physical and physiological variables are obtained (Bulte et al., 2012, Gauthier and Hoge, 2011). These methods have the potential to provide distinct physiological information in conditions where cerebral blood flow and metabolism may be affected. Since Alzheimer's disease (AD) is believed to be associated with both vascular and metabolic effects (Kivipelto et al., 2001, Coskun et al., 2012), it presents a compelling target to study using calibrated fMRI. The objective of the present study was to apply an approach we have previously termed Quantitative O_2_ (QUO2) (Gauthier and Hoge, 2011) in cohorts of probable AD patients and age-matched controls.

There is evidence for mitochondrial dysfunction and oxygen hypometabolism as being a causal factor in AD, as well as in other conditions such as Parkinson's Disease (Bonda et al., 2010, Coskun et al., 2012, Reddy and Beal, 2005, Silva et al., 2011, Sullivan and Brown, 2005, Wallace, 2005). Hence, the QUO2 method allows us to study the hypothesis in which metabolism and vascular dysfunction appear earlier in the disease than cholinergic deficits, beta-amyloid deposition and hyperphosphorylated tau pathology. Moreover, the method may be employed to deepen our understanding of other conditions where vascular and oxygen metabolism may be compromised (such as in Parkinson's Disease). Finally, the physiological information offered by the calibrated fMRI method may be better correlated with the clinical profile of AD than the other biomarker candidates.

While CBF and CMRO_2_ can be quantitatively imaged using positron emission tomography (PET), the use of calibrated fMRI offers several unique features. First, the estimates of CBF and CMRO_2_ from calibrated fMRI are expressed within a biophysical framework that is directly relevant to task activation or resting-state BOLD fMRI studies that are performed in AD (Atri et al., 2011, Belleville et al., 2011, Miettinen et al., 2011, Greicius et al., 2003, Greicius et al., 2004, Gusnard et al., 2001). Any disease-related difference in parameter estimates from calibrated fMRI may thus be readily evaluated in terms of its impact on standard BOLD methods, which may in turn help identify (or rule out) bias associated with underlying physiological changes that are not accounted for in qualitative BOLD analysis.

Calibrated fMRI also provides, either through intermediate results or incidentally, physiological information that is not available through other methods. The QUO2 method used in the present study yields estimates of resting CMRO_2_, oxygen extraction fraction (OEF) and *M* in addition to the transverse relaxation rate constant R2*, and CO_2_ cerebrovascular reactivity (CVR) (expressible in terms of several parameters including CBF, BOLD signal, and R2*). *M* is a sensitivity measure for BOLD fMRI, helping validating the use of the BOLD method as a comparative marker between two groups. R2* is a measure similar to susceptibility-weighted imaging (SWI), it can reflect levels of iron in the tissue as well as level of deoxygenated blood. OEF allows assessment of the presence of cerebral ischemia. These parameters are likely to be influenced by vascular function and oxygen transport, making them potential biomarkers of interest in AD.

Finally, the equipment needed to perform calibrated fMRI techniques may be more readily available for many institutions than equipment necessary to carry out PET radiotracer studies, particularly with short-lived isotopes such as ^15^O. This would facilitate the implementation of multi-center research such as large-scale cohort studies or clinical drug trials.

## 2 Methodology

### 2.1 Participants

A cohort of 65 individuals with mild to moderate dementia of Alzheimer's type and 61 age-matched controls were recruited for the present study. Recruited patients met the criteria for “probable AD dementia” as specified in the NIA/AA Guidelines (McKhann et al., 2011) and had a Mini-Mental State (MMSE) score between 18 and 27. All participants gave written informed consent and the project was approved by the Comité mixte d'éthique de la recherche du Regroupement Neuroimagerie/Québec. Exclusion criteria at entry included: cessation of education before achieving basic literacy in native language, prior history of significant psychiatric or neurological illness (other than AD), significant intellectual handicap, undergoing current or recent (≤ 2 months) treatment for a significant medical condition, chronic obstructive pulmonary disease, history of recurrent asthma, or other significant respiratory disorder, comorbidity with other underlying pathologies for dementia (for AD cohort) and clinical history of cognitive or memory impairment (for control cohort). All participants were either English or French speakers and had a normal or corrected-to-normal hearing and vision (relevant for behavioral testing, although corrective measures were not required during the MRI protocol).

**Fig. 1:**
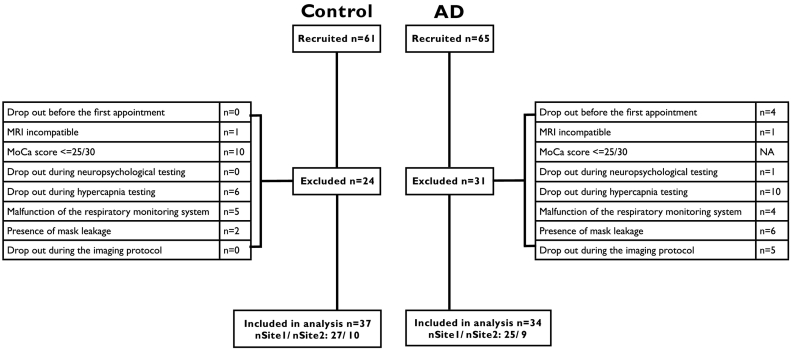
Participant retention. Exclusion criteria for the Alzheimer's patients (AD) and healthy controls cohorts. *MoCa* = Montreal Cognitive Assessment.

From the recruited group, 31 AD and 24 control participants were not included in the present data analysis due to data inclusion criteria outlined in Fig. 1, yielding a final dataset of 34 AD (15 males, mean age of 76.9 ± 6.5) and 37 controls (14 males, mean age of 74.4 ± 4.6). All participants underwent a blood draw, which allowed measurement of their hemoglobin concentration [Hb] that was used to compute CMRO_2_. Participants also completed the Montreal Cognitive Assessment (MoCa) to ensure the absence of cognitive impairment in the control cohort. Control participants who scored < 26 in the MoCa were excluded from the present analysis (n = 10).

### 2.2 Respiratory paradigm

A gas timing schedule previously described by Bulte et al. (2012), with a total duration of 18 min was applied. This involved two 2-minute periods of hypercapnia (HC) and two 3-minute periods of hyperoxia (HO), induced by administering gas mixtures enriched with CO_2_ (5%) and O_2_ (50%) respectively. Hypercapnia was followed by a 1-minute normocapnic period while hyperoxia was followed by a 3-minute period of normoxia. Participants inhaled the gas mixtures via a breathing circuit developed in-house and described by Tancredi et al. (2014). Respiratory gases were continuously monitored using the CO2100C and O2100C modules of a BIOPAC MP150 system (BIOPAC Systems Inc., CA, USA). For additional details, see Lajoie et al. (2016).

Prior to the imaging session, all participants underwent a hypercapnic manipulation of 2 min outside of the scanner for familiarization. Thereafter, participants were interviewed to evaluate their level of respiratory discomfort and to confirm their willingness to continue. Participants who reported a significant discomfort were not invited to continue with the imaging session of the study (10 in the AD group, 6 in the control group).

### 2.3 Image acquisition

Acquisitions were performed on two different clinical 3 T scanners of identical make and model (Siemens TIM TRIO, Siemens Medical Solutions, Erlangen, Germany), using a 32-channel receive-only head coil supplied with this system. It was ensured that an equal number of participants from each group were scanned at each site (Table 1, Pearson Chi-Square *P* value of 0.96). Hence, no adjustment for different sites was performed during the statistical analysis. Participants were asked not to consume caffeine or tobacco for 2 h prior to their imaging session, to avoid confounding vasoactive effects.

**Table 1:** Demographic and clinical data for each group.

	Control	AD	*P* value
Number of subjects	37	34	–
Sites (#1/#2)	27/10	25/9	*P*^a^ = 0.96
Gender (male/female)	14/23	15/19	*P*^a^ = 0.59
Age (years)	74.4 ± 4.6	76.9 ± 6.5	*P*^b^ = 0.07
Education	16.4 ± 3.5	14.6 ± 4.6^c^	*P*^b^ = 0.09
Mini-Mental State (MMSE)^d^	–	23.7 ± 2.6	–
Montreal Cognitive Assessment (MoCa)	28.5 ± 1.2	15.6 ± 5.0	*P*^b^ < 1 × 10^− 14^

Data are means ± standard deviations.

a Pearson Chi-Square *P* value.

b Two-sided independent sampling Student *t*-test *P* value.

c Education information was missing in five AD patients.

d Performed in AD prior to the current study; was part of the inclusion criteria.

The scan session included a 5-minute anatomical acquisition (1 mm^3^ MPRAGE with TR/TE/flip angle = 2.3 s/3 ms/9°, 256 × 240 matrix, GRAPPA factor = 2), and an 18-minute functional scan using a dual-echo pseudo-continuous ASL sequence (de-pCASL) (Dai et al., 2008) in order to acquire simultaneous measures of BOLD and CBF. The de-pCASL parameters were: TR/TE1/TE2/alpha = 4.12 s/8.4 ms/30 ms/90°, labeling duration = 2 s using Hanning window-shaped RF pulse with duration/space = 500 μs/360 μs, flip angle = 25°, slice-selective gradient = 6 mT/m, label offset = 100 mm below the center of image slab, nominal and average post-labeling delay (PLD) = 0.9 and 1.44 s respectively. The readout consisted of a GRE-EPI with GRAPPA factor = 2, partial sampling of k-space = 7/8, in-plane resolution of 4.5 × 4.5 mm^2^, 21 slices with 4.5 mm thickness and 0.5 mm gap.

### 2.4 Respiratory data analysis

Analysis of the respiratory data was carried out using a custom software application developed in Matlab (MathWorks, Natick, MA, USA), which performed automatic determination of the end-expiratory (end-tidal (ET)) and end-inspiratory points from the continuous O_2_ and CO_2_ sampling. Each ET point was corrected to account for the low-pass filtering effect of the filter placed in series and to account for an expired partial pressure of water of 47 mmHg (Severinghaus, 1989). For more details, see Lajoie et al. (2016).

The average values of ETO_2_ at baseline and during both respiratory stimuli were used to compute arterial O_2_ content (ml O_2_/ml blood) as well as the change in venous deoxygenated fraction ([dHb]/[dHb]_0_), as detailed by Chiarelli et al. (2007) and Gauthier et al. (2012). The latter quantities are needed to obtain the BOLD calibrated value *M*, resting OEF and CMRO_2_ as specified below.

### 2.5 Image data preprocessing

Analysis of functional scans was performed using in-house software implemented in C, as in Lajoie et al. (2016). Susceptibility artifacts caused by the paramagnetic molecular O_2_ that was inspired, compounded with the thick slices used to maximize SNR, limited the image quality of regions on the ventral surface of the brain and those adjacent to the nasal cavity. These regions were excluded from the analysis by empirically determining a threshold in the intensity normalized S0 (label and control averaged) that delineate them. The ASL signal was converted into physiological units of flow (ml/100 g/min) as in Wang et al. (2003) with a blood-brain partition coefficient = 0.9, labeling efficiency = 0.85, blood T_1_ = 1.65 s, grey matter T_1_ = 1.4 s (Alsop et al., 2015) and an adjusted PLD to account for slice acquisition time (PLD range for 21 slices of 900–1960 ms). The control S0 at baseline was used as a surrogate for the fully relaxed magnetization M0. In order to compensate for incomplete recovery of longitudinal magnetization during our TR of 4.12 s, a factor implicating the grey matter T_1_ value was applied to the baseline EPI estimates (Alsop et al., 2015).

In general, the CBF response to hyperoxia is known to be small (Bulte et al., 2006), but small regional changes are difficult to measure due to the low SNR of ASL. Because of this, a uniform change in CBF was assumed throughout the brain, based on averaging of global responses over all participants (the ultimate effect on accuracy is minimal due to the small effect size) (Gauthier et al., 2012, Chiarelli et al., 2007, Bulte et al., 2012, Bulte et al., 2006). The result of averaging all participants' grey matter ∆%CBF_HO_ corrected for blood T_1_ was − 2%, as described next. During hyperoxic manipulation, the T_1_ of blood is altered due to an increase in plasma concentration of paramagnetic O_2_ (Pilkinton et al., 2011). To account for this change in blood T_1_, which biases the measured CBF changes, a corrective factor using the approach described in Chalela et al. (2000) and Zaharchuk et al. (2008) was applied. First, arterial blood T_1_ values during hyperoxic periods were linearly interpolated based on the individual ETO_2_ measurements, used as a surrogate for arterial partial pressure of O_2_ (PaO_2_), along with the R1 (1/T_1_) and PaO_2_ relationship in rats' blood reported by Pilkinton et al. (2011). Then, the individual blood flow maps during hyperoxia were corrected by applying a slice-wise corrective factor based on the quantitative blood flow equation (Wang et al., 2003), slice acquisition time and adjusted T_1_ value.

### 2.6 Computation of CMRO_2_

For each gas challenge, the changes in the venous deoxygenated fraction, along with the changes in BOLD (∆R2*) and CBF were used as inputs to the generalized calibration model (GCM), described in Gauthier and Hoge (2012). This yields a system of two equations with two unknowns: the BOLD calibration parameter *M* (extrapolated maximum BOLD fractional signal increase when venous O_2_ saturation approaches 100%) and OEF (the fraction of delivered oxygen that is consumed). Absolute CMRO_2_ was then determined by multiplying OEF by O_2_ delivery, computed as the product of resting CBF by arterial O_2_ content. In the absence of an intersection between the hypercapnia and hyperoxia curves, the voxel is determined to have no solution and will be excluded from any further analysis. In the equation defining *M* (Gauthier and Hoge, 2012, equation 7), the parameter α, which expresses the relationship between changes in blood flow and blood volume, was assumed to be 0.18 (Chen and Pike, 2010b) while β, defining the non-linear dependence of changes in R2* on deoxygenated hemoglobin, was set to 1.5 (Boxerman et al., 1995). The hemoglobin concentration [Hb] was measured from blood drawn from each participant during the scanning session. Metabolic changes associated with periods of hypoxia and hypercapnia remain a topic of debate (Chen and Pike, 2010a, Hino et al., 2000, McPherson et al., 1991, Zappe et al., 2008, Xu et al., 2010, Xu et al., 2012). Without a clear consensus on the matter, isometabolism was assumed as in previous calibrated studies (Lajoie et al., 2016, Gauthier et al., 2013, Gauthier et al., 2012).

### 2.7 Statistical analysis

#### 2.7.1 Patients versus healthy control subjects

Regional-based analysis was performed in each individual's original image space (i.e. no spatial resampling). Regions of interest (ROIs) included bilateral grey matter regions for each of the four lobes, as defined by the ICBM152 lobes atlas (Fonov et al., 2011b), which was non-linearly registered to the ADNI template (Fonov et al., 2011a): frontal, parietal, temporal and occipital, as well as two sub-regions known to be vulnerable to AD (Buckner, 2005), i.e. posterior cingulate and precuneus defined from the OASIS-TRT-20 atlas (Klein and Tourville, 2012). Given the low SNR in ASL, averaging within lobes was a reasonable compromise in terms of specificity and sensitivity, while allowing for coverage of most of the data and avoided the more arbitrary definition of smaller regions. A region including all four lobes was also created to evaluate measurements throughout cerebral grey matter. A probability mask of grey matter was automatically extracted from T_1_-weighted scans using the FMRIB Software Library (FSL) (Jenkinson and Smith, 2001), then resampled to the functional EPI scans. Each ROI was also registered to the resolution of the functional EPI scans before being further masked using the individual's grey matter probability map to exclude voxels with a grey matter probability lower than 50%. The ROIs and grey matter probability maps were then used to compute weighted averages for each metric. This weighting procedure served to account for the partial volume effect due to the presence of a mixture of grey matter, white matter and cerebrospinal fluid in EPI voxels. Missing data due to differences in imaged slice positions, susceptibility patterns, or no-solution patterns for *M* and OEF, were not included in the average. ROI analyses between groups were performed using Matlab (Mathworks, Natick, MA) by applying a univariate general linear model with *P* < 0.05 level of significance. Since age is known to be a predominant factor in cerebral hemodynamics changes (De Vis et al., 2015, Gauthier et al., 2013), results were obtained with and without adjustment for age.

A voxel-wise analysis was also performed to corroborate results of the ROI-based analysis. Individual maps were non-linearly registered, with 12 degrees of freedom, to the ADNI atrophy-specific MRI brain template created from a subset of cognitively normal subjects, patients with mild cognitive impairment (MCI) and patients with mild AD dementia (Fonov et al., 2011a). The non-linear registration was calculated using the CIVET software package (Collins et al., 1995) via the CBRAIN interface (Sherif et al., 2014). Brain coverage in normalized group-average data was somewhat limited due to inter-individual differences in: 1) image volume coverage, 2) patterns of susceptibility artifacts, and 3) no-solution patterns for *M* and OEF. To help correct for these effects, missing data within each participant was interpolated to the respective group-average value and the analyzed region only included voxels where a minimum of 20 participants per group had a valid value. Parametric images were then tested for significant difference between groups by performing a grey matter voxel-wise analysis using SPM12, including age as a covariate. Statistical parametric maps were assessed for cluster-wise significance using a cluster-defining threshold of *P* = 0.005 (uncorrected), while the 0.05 FWE-corrected critical cluster size was set to a minimum of 1250 voxels (voxels of 1x1x1mm^3^). If needed, the cluster size was adjusted to eliminate clusters that were not large enough to reach significance according to the SPM model. A toolbox for SPM was employed to identify the location of clusters, by calculating the percentage of cluster within each region (Tzourio-Mazoyer et al., 2002). The two highest percentages per cluster are reported.

#### 2.7.2 Correlation between QUO2 findings and MoCa scores in AD

Correlations between Montreal Cognitive Assessment (MoCa) scores and our imaging findings in AD were tested for significance using Spearman's rank-order correlations, with statistical significance set at *P* 0.05. Correlation with MMSE scores was not performed since these tests were obtained prior to the present study.

## 3 Results

### 3.1 Participant demography

The demographic characteristics of the participants are shown in Table 1. We obtained a very good segregation between the two groups based on their respective Montreal Cognitive Assessment (MoCa) score. The difference in age between our groups neared significance, which supports the need to correct for its effect on the different hemodynamic parameters.

### 3.2 Susceptibility artifact

As expected, with the presence of paramagnetic molecular oxygen in inhaled air situated in the frontal sinuses and nasal cavity, the temporal lobe was the main region in which voxels were excluded due to susceptibility artifact: a reduction of 4% in the number of voxels (having a minimal grey matter probability of 50%) was observed. The number of voxels in the occipital and frontal lobes reduced by < 1%, while the parietal lobe was not affected.

### 3.3 Delayed arterial transit time

**Fig. 2:**
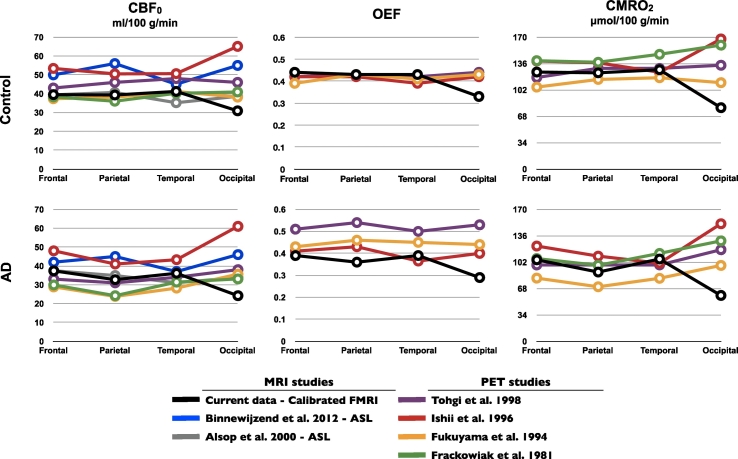
Bias in occipital lobe due to delayed arterial transit time (ATT). Our resting CBF, OEF and CMRO_2_ values within each grey matter lobe are compared to those found in the literature. Measurements fall within the range of reported values, excepting in the occipital where the ATT is suspected to be longer than our time acquisition, biasing our ASL measurements, and thus the derived-model *M*, OEF and CMRO_2_ estimates in this region. *CBF*_*0*_ = the resting oxygen delivery; *OEF* = the oxygen extraction fraction; *CMRO*_*2*_ = the resting oxygen consumption.

We compared resting CBF, OEF and CMRO_2_ values within the four grey matter lobes (Fig. 2) with that of previous ASL (Binnewijzend et al., 2013, Alsop et al., 2000) and PET studies (Tohgi et al., 1998, Ishii et al., 1996b, Fukuyama et al., 1994, Frackowiak et al., 1981). Our values are in good general agreement with previous studies, except in the occipital lobe where our estimates are lower in both groups. The occipital lobe is served by the posterior cerebral artery, which has a substantially longer transit time (MacIntosh et al., 2010, Mutsaerts et al., 2015, Donahue et al., 2014). Therefore, we suspect our CBF estimates to be biased in this area only, due to a short PLD. Conjointly, higher CBF delta percent change, during hypercapnia, in the occipital lobe compared with the other lobes was also observed and supports this theory (data not shown). This bias in resting CBF and in flow change to hypercapnia propagates to the model-derived *M*, OEF and CMRO_2_, which prevents us from making any conclusions in the occipital lobe. Therefore, we chose not to include the occipital lobe in subsequent analyses.

### 3.4 Patients versus healthy control subjects

#### 3.4.1 Respiratory data

**Fig. 3:**
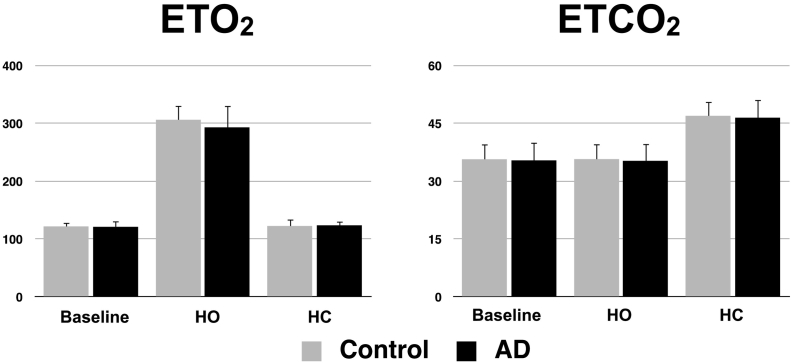
Gas manipulations. End-tidal (ET) O_2_ and CO_2_ values at baseline, during hyperoxia (HO) and during hypercapnia (HC) for both groups. Errors bars indicate standard deviation.

Fig. 3 shows the O_2_ and CO_2_ end-tidal values (ET) at baseline, during hyperoxia (HO) and hypercapnia (HC) in both groups. No significant differences in respiratory measurements were found between the groups (P_ETO2_0_ = 0.5, P_ETO2_HO_ = 0.2, P_ETO2_HC_ = 0.8, P_ETCO2_0_ = 1.0, P_ETCO2_HO_ = 1.0, P_ETCO2_HC_ = 0.8, adjusted for age).

#### 3.4.2 Hemoglobin concentration and T_1_corrected CBF change during hyperoxia

The blood test revealed no significant difference in the hemoglobin concentration ([Hb]) between groups (*P* = 0.4 adjusted for age), with an average of 13.9 ± 1.4 and 13.9 ± 1.2 gHb/dl in the patient and healthy control cohorts, respectively. Individual values of [Hb] were used in the QUO2 model for M, OEF, and CMRO_2_ estimation. Blood T_1_ during hyperoxia was estimated to be 1.593 ± 0.015 and 1.587 ± 0.011 s in AD and controls respectively. These were not significantly different (*P* = 0.2, adjusted for age). As a result, the grey matter group-averaged T_1_-corrected CBF values during hyperoxia were − 1.7 ± 10.9 and − 2.6 ± 8.9% in AD and controls respectively, with no significant difference between them (*P* = 0.9, adjusted for age). Considering the low SNR associated with these measurements, the T_1_-corrected ∆%CBF_HO_ averaged over both groups (− 2%) was employed as a fixed constant in the computation of the model-derived estimates (descriptions in Methodology section).

#### 3.4.3 Region-wise analysis

Our grey matter region-wise analysis revealed a decreased CBF and CMRO_2_ in the parietal, precuneus and temporal regions of AD patients compared to age-matched controls (Table 2). Interestingly, all regions showed a lower resting R2* in AD (corresponding to a slower transverse relaxation of the MRI signal). The R2*_0_ in AD was also found significantly lower in all investigated regions. During the hypercapnic manipulation, R2* decreased less in AD (equivalent to a lower BOLD signal increase), in the frontal, parietal, and precuneus regions, as well as the whole brain. A tendency for a reduced R2* decrease during hypercapnia was observed in the temporal and posterior cingulate cortex, although the differences were not significant after adjustment for age (*P* = 0.07 and *P* = 0.06, respectively). Lower OEF values in the parietal, precuneus and the whole brain of AD were not significant after adjustment for age. No region showed a significant difference between groups for the *M* value, the R2* change during hyperoxia, and the CBF change during hypercapnia. Since the increase in end-tidal CO_2_ during hypercapnia was equivalent in both groups, the percent CVR was also not found to be significantly different.

**Table 2:** Region-wise analysis in grey matter with and without adjustment for age. For each physiological variable, group average values ± standard deviation in different ROIs are reported, as well as Student *t*-test *P* values calculated with and without adjustment for age. *P* values where significance is reached (*P* < 0.05) are shown in bold. Physiological variables are: CBF_0_ (ml/100 g/min), the resting oxygen delivery; OEF, the oxygen extraction fraction; CMRO_2_ (μmol/100 g/min), the resting oxygen consumption; *M* (%) the maximum BOLD signal increase when venous O_2_ saturation approaches 100%; R2*_0_ (s^− 1^), the transverse relaxation rate constant; ∆%CBF_HC_ (%), the blood flow percent change during hypercapnia; %CVR (%), the cerebrovascular reactivity in percent blood change to change in end-tidal CO_2_; ∆R2*_HC_ (s^− 1^), the R2* change during hypercapnia; ∆R2*_HO_ (s^− 1^), the R2* change during hyperoxia.

		Frontal	Parietal	Temporal	Precuneus	Posterior cingulate	Total^c^
CBF_0_	Control	39.5 ± 10.8	39.3 ± 13.1	41.2 ± 10.8	37.2 ± 14.4	43.7 ± 11.4	39.8 ± 11.2
	AD	37.4 ± 9.0	32.7 ± 9.8	36.1 ± 8.3	29.2 ± 10.5	39.9 ± 8.6	35.9 ± 8.8
	*P*^a^/*P*^b^	0.37/0.29	**0.02/0.02**	**0.03/0.01**	**0.01/0.007**	0.12/0.08	0.11/0.07
OEF	Control	0.44 ± 0.14	0.43 ± 0.14	0.43 ± 0.13	0.42 ± 0.16	0.47 ± 0.15	0.43 ± 0.13
	AD	0.39 ± 0.08	0.36 ± 0.10	0.39 ± 0.10	0.34 ± 0.13	0.45 ± 0.16	0.38 ± 0.08
	*P*^a^/*P*^b^	0.06/0.13	**0.02**/0.08	0.16/0.33	**0.04/**0.12	0.63/0.72	**0.03/**0.09
CMRO_2_	Control	125 ± 44	124 ± 48	128 ± 40	113 ± 55	144 ± 57	124 ± 42
	AD	105 ± 31	89.2 ± 33.6	106 ± 32	75.6 ± 39.2	130 ± 59	99.9 ± 28.6
	*P*^a^/*P*^b^	**0.04/**0.09	**0.001/0.004**	**0.01/0.04**	**0.002/0.007**	0.32/0.37	**0.007/0.02**
*M*	Control	5.11 ± 1.14	5.79 ± 1.95	6.59 ± 1.69	6.32 ± 2.58	6.20 ± 2.32	5.62 ± 1.33
	AD	4.92 ± 1.53	5.12 ± 1.77	6.43 ± 1.39	5.35 ± 1.93	6.32 ± 3.35	5.24 ± 1.36
	*P*^a^/*P*^b^	0.55/0.82	0.14/0.36	0.68/0.76	0.08/0.22	0.86/0.73	0.25/0.57
R2*_0_	Control	18.0 ± 1.4	17.2 ± 1.5	24.9 ± 1.4	16.9 ± 1.6	16.2 ± 1.1	19.6 ± 1.3
	AD	16.8 ± 1.0	15.9 ± 1.4	24.1 ± 1.4	15.7 ± 1.4	15.1 ± 1.4	18.4 ± 1.1
	*P*^a^/*P*^b^	**1 × 10**^**–4/**^**/5 × 10**^**− 4**^	**3 × 10**^**− 4**^**/2 × 10**^**− 3**^	**3 × 10**^**− 3**^**/5 × 10**^**− 2**^	**6 × 10**^**− 4**^**/3 × 10**^**− 3**^	**2 × 10**^**− 4**^**/9 × 10**^**− 4**^	**1 × 10**^**− 4**^**/7 × 10**^**− 4**^
∆%CBF_HC_	Control	53.8 ± 22.8	63.3 ± 49.7	71.8 ± 39.0	69.5 ± 59.6	37.0 ± 27.9	61.0 ± 30.9
	AD	46.5 ± 23.3	62.4 ± 42.6	62.0 ± 33.0	65.3 ± 44.1	29.1 ± 24.7	54.5 ± 26.8
	*P*^a^/*P*^b^	0.20/0.29	0.94/0.88	0.27/0.34	0.74/0.61	0.22/0.35	0.35/0.42
%CVR	Control	4.80 ± 1.93	5.45 ± 3.66	6.32 ± 2.87	6.02 ± 4.45	3.24 ± 2.48	5.37 ± 2.31
	AD	5.25 ± 5.47	6.90 ± 7.90	6.34 ± 4.10	7.15 ± 7.66	3.39 ± 4.77	5.91 ± 5.35
	*P*^a^/*P*^b^	0.65/0.72	0.32/0.49	0.98/0.97	0.45/0.71	0.87/0.91	0.58/0.71
∆R2*_HC_	Control	− 0.57 ± 0.19	− 0.77 ± 0.25	− 0.65 ± 0.25	− 0.91 ± 0.30	− 0.62 ± 0.18	− 0.64 ± 0.21
	AD	-0.43 ± 0.18	− 0.62 ± 0.23	− 0.51 ± 0.23	− 0.73 ± 0.29	− 0.50 ± 0.21	− 0.50 ± 0.19
	*P*^a^/*P*^b^	**0.003/0.02**	**0.01/0.04**	**0.02**/0.07	**0.01/0.04**	**0.01**/0.06	**0.004/0.02**
∆R2*_HO_	Control	− 0.15 ± 0.09	− 0.25 ± 0.10	− 0.18 ± 0.13	− 0.28 ± 0.11	− 0.24 ± 0.08	− 0.19 ± 0.09
	AD	-0.17 ± 0.09	− 0.26 ± 0.10	− 0.19 ± 0.13	− 0.29 ± 0.12	− 0.23 ± 0.08	− 0.20 ± 0.09
	*P*^a^/*P*^b^	0.51/0.42	0.66/0.51	0.83/0.59	0.78/0.70	0.74/0.90	0.61/0.44

a Two-sided independent sampling Student *t*-test *P* value, without adjustment for age.

b Two-sided independent sampling Student *t*-test *P* value, with adjustment for age

c Cerebral grey matter excluding the occipital lobe

#### 3.4.4 Voxel-wise analysis

**Fig. 4:**
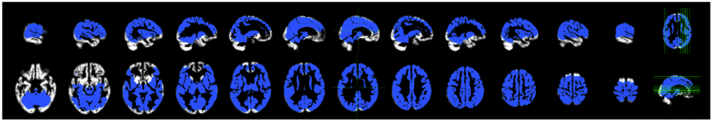
Volume of the brain analyzed. Analyzed volume (in blue) only includes voxels where a minimum of 20 participants per group had a valid value. Any remained missing data within the analyzed region were interpolated to the respective group-averaged value. Missing data are due to differences in: 1) imaged slice position, 2) susceptibility patterns and 3) pattern of no-solutions across subjects. In the axial slices: left = left. (For interpretation of the references to color in this figure legend, the reader is referred to the web version of this article.)

**Fig. 5:**
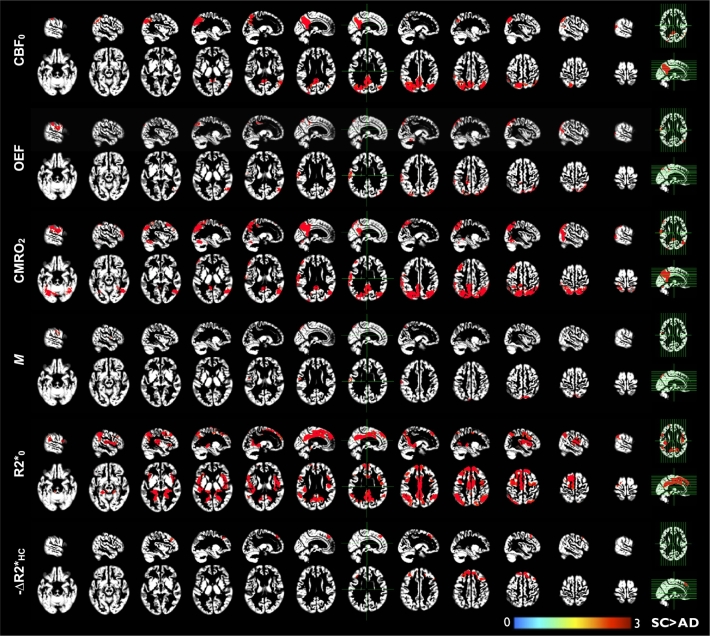
Voxel-based analysis adjusted for age. For each physiological variable, the colored regions show a significant deficit in AD (primary *P* < 0.005 and minimum cluster size of 1250 mm^3^, yielding a FWE cluster-corrected (*P* < 0.05)). The color bar indicates Student's *t*-statistic value. Statistically significant clusters are overlaid to different sagittal and axial sections of the ADNI template. Physiological variables are: CBF_0_, the resting oxygen delivery; OEF, the oxygen extraction fraction; CMRO_2_, the resting oxygen consumption; *M*, the maximum BOLD signal increase when venous O_2_ saturation approaches 100%; R2*_0_, the transverse relaxation rate constant; ∆R2*_HC_, the R2* change during hypercapnia. In the axial slices: left = left. *SC* = subject control. (For interpretation of the references to color in this figure legend, the reader is referred to the web version of this article.)

The brain volumes included in the grey matter voxel-wise analysis is shown in Fig. 4, overlaid on different sagittal and axial sections of the ADNI template. Given the biased ASL signal in the occipital lobe, this lobe was also excluded from the analysis. Clusters with significant differences between groups are reported in Fig. 5 and Table 3. No cluster showed significant increases in AD relative to controls. Significant reductions in CBF were observed in the temporal and parietal lobes in AD, including the precuneus and posterior cingulate. Reductions in CMRO_2_ were observed in the frontal, temporal and parietal lobes, more specifically in the precuneus. Lower OEF and calibrated parameter *M* were also found in the parietal-precuneus, although the sizes of significant clusters were considerably smaller. Also observed in the region-wise analysis, resting R2* was lower in different AD brain regions, such as in bilateral temporal, frontal, parietal, precuneus and posterior cingulate. As for the change in R2* during hypercapnia, only the frontal lobe revealed a significantly smaller decrease in AD relative to controls. Finally, no significant group difference was found for ∆R2*_HO_, ∆%CBF_HC_ and %CVR.

**Table 3:** Clusters with significant difference (control > AD) adjusted for age. For each physiological variable, significant clusters are reported. Physiological variables are: CBF_0_, the resting oxygen delivery; OEF, the oxygen extraction fraction; CMRO_2_, the oxygen consumption; *M*, the maximum BOLD signal increase when venous O_2_ saturation approaches 100%; R2*_0_, the transverse relaxation rate constant; ∆R2*_HC_, the R2* change during hypercapnia.

	Clusters	*N* voxels	MNI coordinates x, y, z mm	Anatomical areas (first two containing the highest % of the cluster)	%
CBF_0_	1	43,012	− 40 − 88 27	Right precuneus	23.85
				Left postcentral	12.37
	2	10,652	49 − 71 39	Right superior temporal	36.97
				Right superior parietal	20.65
OEF	1	10,176	55 − 71 11	Right inferior parietal	25.30
				Right inferior temporal	24.45
	2	4852	− 67 − 14 26	Left inferior parietal	45.98
				Left superior temporal	21.91
	3	2148	− 2 − 76 52	Left superior parietal	23.74
				Left precuneus	19.69
	4	1699	11 − 42 − 32	Right cerebellum	47.32
				Right cerebellum	30.37
	5	1552	− 13 − 40 47	Left precuneus	52.26
				Left supplementary motor area	28.03
CMRO_2_	1	95,725	53 − 67 − 13	Right precuneus	13.16
				Right superior parietal	8.76
	2	2813	− 55 36 8	Left inferior pars triangularis	41.41
				Left middle frontal	7.18
	3	4491	− 25 31 56	Left superior frontal	4.24
				Left middle frontal	3.07
	4	8699	− 29 − 74 − 26	Vermis	50.39
				Left inferior temporal	29.82
*M*	1	2107	8 − 75 54	Right precuneus	28.14
				Left precuneus	24.35
	2	1799	− 67 − 13 33	Left supramarginal	67.54
				Left heschl	12.51
R2*_0_	1	21,132	46 − 9 10	Right postcentral	23.51
				Right insula	18.90
	2	14,888	− 48 6 − 2	Left insula	25.91
				Left superior temporal pole	18.48
−∆R2*_HC_	1	6094	− 9 39 53	Left inferior pars triangularis	35.58
				Left middle frontal	35.12
	2	6115	8 44 50	Right inferior pars opercularis	31.81
				Right middle frontal	29.84

#### 3.4.5 Correlation between QUO2 findings and AD MoCa scores

MoCa scores were only significantly correlated with R2*_0_ in the whole brain grey matter (occipital lobe excluded) and temporal cortex (*r* = 0.37, *P* = 0.04; *r* = 0.36, *P* = 0.04 respectively) (Table 4). MoCa scores correlation with R2*_0_ in the posterior cingulate and CBF_0_ in the precuneus approached significance (*r* = 0.34, *P* = 0.06; *r* = 0.32, *P* = 0.07 respectively).

**Table 4:** Correlation between physiological variables in grey matter and MoCa scores in AD. Values represent the Spearman correlation coefficients (*P* value) between the Montreal Cognitive Assessment (MoCa) scores and the physiological variables in AD. Physiological variables are: CBF_0_, the resting oxygen delivery; OEF, the oxygen extraction fraction; CMRO_2_, the resting oxygen consumption; *M*, the maximum BOLD signal increase when venous O_2_ saturation approaches 100%; R2*_0_, the transverse relaxation rate constant; ∆%CBF_HC_, the blood flow percent change during HC; %CVR, the cerebrovascular reactivity in percent blood change to change in end-tidal CO_2_; ∆R2*_HC_, the R2* change during hypercapnia; ∆R2*_HO_, the R2* change during hyperoxia.

	Frontal	Parietal	Temporal	Precuneus	Posterior cingulate	Total^a^
CBF_0_	− 0.05 (0.79)	0.20 (0.28)	0.29 (0.11)	0.32 (0.07)	− 0.04 (0.84)	0.06 (0.75)
OEF	− 0.05 (0.80)	0.12 (0.50)	− 0.03 (0.85)	0.12 (0.53)	− 0.23 (0.20)	0.04 (0.83)
CMRO_2_	− 0.01 (− 0.94)	0.20 (0.28)	0.12 (0.50)	0.29 (0.10)	− 0.24 (0.19)	0.05 (0.77)
*M*	− 0.05 (0.80)	0.06 (0.74)	0.00 (0.98)	0.15 (0.41)	− 0.21 (0.25)	0.02 (0.90)
R2*_0_	0.25 (0.16)	0.22 (0.22)	0.36 (0.04)⁎	0.16 (0.39)	0.34 (0.06)	0.37 (0.04)⁎
∆%CBF_HC_	− 0.14 (0.45)	− 0.17 (0.34)	− 0.14 (0.42)	− 0.13 (0.49)	0.08 (0.65)	− 0.16 (0.38)
%CVR	− 0.14 (0.46)	− 0.18 (0.34)	− 0.16 (0.38)	− 0.12 (0.51)	0.1 (0.59)	− 0.21 (0.25)
∆R2*_HC_	0.12 (0.51)	− 0.03 (0.87)	0.14 (0.45)	− 0.06 (0.73)	− 0.02 (0.93)	0.09 (0.63)
∆R2*_HO_	0.09 (0.61)	0.08 (0.68)	− 0.04 (0.82)	0.09 (0.62)	0.01 (0.96)	0.09 (0.62)

⁎ *P* < 0.05.

a Cerebral grey matter excluding the occipital lobe.

## 4 Discussion

In this study, we investigated whether our QUO2 technique was sensitive to differences in vascular and metabolic function in AD. This calibrated fMRI technique has the advantage of providing quantitative information while being readily integrated with other MRI measures (i.e. structural MRI) of proven value in AD, and may therefore offer new insights into the underlying mechanisms and causes of the disease.

Although the QUO2 model-derived parameters and MoCa scores in AD were not significantly correlated, vascular and metabolic deficits in AD may be associated with other cognitive scores including: language, verbal reasoning, visuospatial function, as well as visual, short-term and working memory. Correlations between these neuropsychological scores and our imaging findings will be evaluated in a subsequent paper.

### 4.1 Patients versus controls

The hypoperfusion in AD observed in our data supports its relevance as a pathophysiological change in AD (Iturria-Medina et al., 2016, Aliev et al., 2003, de la Torre, 2002) and is consistent with findings from a previous ASL studies (Alsop et al., 2000, Alsop et al., 2008, Dai et al., 2009, Binnewijzend et al., 2013). The absence of significant differences in the frontal lobe blood flow (39.5 ± 10.8 vs. 37.4 ± 9.0 ml/100 g/min) is consistent with an AD-like pattern, as opposed to vascular dementia (Nagata et al., 2000). With a power of test of 80%, our minimum detectable difference (MDD) in frontal lobe blood flow was 7 ml/100 g/min. Our data showed a coupled parietotemporal pattern of hypoperfusion and hypometabolism in AD, which corresponds to findings in previous PET studies (Ishii et al., 1996a, Nagata et al., 2000, Tohgi et al., 1998), including changes in the precuneus, an area where blood flow and glucose use was shown to decline at a very early stage in AD (Asllani et al., 2007, Binnewijzend et al., 2013, Dai et al., 2009, Langbaum et al., 2010, Sakamoto et al., 2002). Although the coupling of blood flow and metabolism in AD may imply that the CBF measurement alone is a reliable way of looking at cerebral function, evidence for some decoupling was previously reported (Nagata et al., 1997), hence, both measurements remain pertinent especially when exploring the causal factors of the disease or assessing response to treatment. For instance, by comparing the MRI oxygen uptake to the PET FDG glucose uptake, one could compute a brain glycolytic index that would flag regions where the oxygen metabolism, and thus indirectly the mitochondrial function, is being compromised and may contribute to the progression of the disease (Bonda et al., 2010, Coskun et al., 2012, Reddy and Beal, 2005, Sullivan and Brown, 2005, Wallace, 2005).

Our voxel-wise analysis on OEF indicated a significant decrease in the AD cohort, within the parietal lobe, while our region-wise analysis reported no significant differences after correction for age (MDD_Total_ = 0.09). Results in the literature are inconsistent regarding OEF, with some studies failing to find a difference (Fukuyama et al., 1994, Frackowiak et al., 1981), others noting a decrease in the medial temporal (Ishii et al., 1996b), and still others suggesting an increase in the parietal cortex (Tohgi et al., 1998). The coupled reduction in CBF and CMRO_2_ along with the absence of increased OEF tends to rule out chronic global cerebral ischemia in our AD patients, at least at this stage of the illness.

A trend toward lower calibrated BOLD *M* values in aging has been previously observed (De Vis et al., 2015, Gauthier et al., 2013, Mohtasib et al., 2011, Ances et al., 2009), while, to our knowledge, this study represents the first determination of the *M* value in an AD cohort. The *M* value is determined largely by the baseline deoxyhemoglobin (dHb) brain content. If resting dHb content in the brain is different between two groups, standard BOLD measures must be interpreted with caution, since a task-related BOLD signal increase will depend partly on the baseline dHb. In the present study, we found few differences between groups on a voxel-wise basis and no significant difference was found between regions (MDD_Total_ = 0.9%). This finding tends to support the use of BOLD as a comparative biomarker between the two groups studied.

The observed reduction in transverse relaxation rate constant in AD relative to control was unexpected. Iron, by its paramagnetic nature can increase the R2* effect and it is known to be higher in AD, most notably in sub-cortical regions and hippocampus (Stankiewicz et al., 2007), which were not considered in the present analysis. The presence of more brain atrophy in AD (Jack et al., 1999, Fox et al., 1999, Josephs et al., 2008, Schott et al., 2008, Sluimer et al., 2010) might explain a lower R2* due to the greater inclusion of cerebrospinal fluid in our voxel-wise and regional analyses. In the present study, we sought to limit such partial volume errors by weighting our ROI-averaging with the maps of grey matter fraction determined from individual T_1_-weighted images. Otherwise, lower R2* in AD may be due to less deoxygenated hemoglobin or a better shim due to the grey matter being further away from scalp (due to atrophy).

Another interesting finding is the absence of significant differences in percent CVR between our AD and control groups (MDD_Total_ = 1.56%). Nagata et al., 1997, Nagata et al., 2000 made the same observation while comparing the vascular reactivity to carbon dioxide inhalation between a group of probable AD, a group of patients with vascular dementia (VaD) and a group of age-matched controls. They found that while the CVR was depleted in the VaD group, it remained normal in the AD group. Our results are consistent with the latter finding, suggesting physiological changes specific to AD rather than vascular dementia.

### 4.2 Limitations

Analyses were performed solely on the basis of the participant's clinical diagnostic status, without taking into account vascular lesion burden that may play a role in the observed results. We will follow up on this in a subsequent paper, while taking into account the fluid-attenuated inversion recovery (FLAIR) and susceptibility-weighted imaging (SWI) scans that were acquired during the MRI session.

During the hypercapnic testing, 10 out of 59 patients (17%) and 6 out of 60 controls (10%) withdrew due to the feeling of anxiety caused by the CO_2_ inhalation. Previous studies of respiratory manipulation in our research group (Lajoie et al., 2016, Gauthier et al., 2013) showed a lower rate of withdrawal associated with periods of hypercapnia. However, the previous study included only healthy participants in their sixties (64 ± 5 years) or younger (31 ± 6 years), the present participants were in their seventies (group-average age of 76 ± 6 years). Younger participants are, understandably, more comfortable with the procedure than elderly participants and AD patients. It is likely that a clinical trial would experience a lower rate of dropouts among a cohort of mild cognitive impairment (MCI) patients than within a group of AD patients. Also, dropouts were apparent predominantly in the beginning of the study, decreasing from 17% of participants in the first half of the study to 10% of participants in the second half. As time passed, study coordinators got better at identifying potential participants who, by virtue of cognitive or other deficits, were less likely to tolerate the protocol (although this admittedly constitutes a selection bias). Moreover, the testing protocol (which included a blood test, neuropsychological battery, hypercapnia test and MRI scan) was performed during a single visit. Future calibrated studies may consider planning for a separate day to perform the hypercapnia testing alone, thus reducing potential stress and fatigue experienced by the participants.

Our cohorts' size was also diminished due to either the presence of mask leakage or malfunction of the monitoring system (which affected data for 10 patients and 7 controls), which may be seen as problematic in the context of a trial. Malfunction of the monitoring system consisted of a failing O_2_ pump at some point during the first half of the study, and can be considered as an isolated event since no more difficulties were observed following its repair. The leaks were either due to an imperfect fit of the mask to the participant's face, or small fissures in the valves of the gas system circuit. The fissures in the valves were the result of a manufacturing defect in a specific batch of breathing circuits. Hence, it is unlikely that future clinical trials would experience the same challenge. Also, as time passed, experimenters became experienced at identifying leaks during the acclimation task before the imaging session and hence were able to fix it by either re-positioning the mask, employing skin tape to eliminate any remaining apertures or replacing the defective valve. Data exclusion due to technical challenges passed from 22% in the first half of the study to 5% in the second half.

Although our detectable effect is limited by our final cohort sizes (AD = 34, Control = 37), which in turn limits the statistical power, the clear segregation of the two groups based on their MoCa score (15/30 vs. 28/30) suggests that the observed effects are specific to AD.

Our lower CBF_0_ and higher ∆%CBF_HC_ found in the occipital lobe are indicative that for a majority of participants, our post-labeling-delay (PLD) was insufficient due to the longer transit time of the artery serving the occipital lobe. When quantifying the blood flow, as long as the PLD is equal or higher than the time taken by the blood bolus to travel from the labeled slice to the imaged tissue, or arterial transit time (ATT), the exact value of the latter does not matter since the CBF measurement will not be biased by incomplete label delivery. The ATT is known to lengthen with age (Liu et al., 2011, Mutsaerts et al., 2015), to vary within brain regions served by different major cerebral arteries (MacIntosh et al., 2010, Mutsaerts et al., 2015, Donahue et al., 2014), to differ between grey matter and white matter, and to decrease under hypercapnic manipulation (Donahue et al., 2014). Since the ASL signal decays rapidly with time, employing a PLD value high enough to overtake the ATT under all conditions, participants and regions, would be costly in terms of SNR. Therefore, the choice of PLD is a compromise between SNR, sensitivity and specificity. In this study, we opted for a moderate PLD ranging from 900 ms and 1986 ms (given the different slice acquisition times), resulting in a brain averaged PLD of 1443 ms, which is lower than the whole brain PLD of 2000 ms later recommended by the ASL community (Alsop et al., 2015). Our decision was based on 1) the presence of a hypercapnic manipulation in our study, which is known to accelerate the blood flow and therefore to lower the ATT, 2) the loss of SNR that we would experience in the upper slice if increasing the whole range of PLD values and 3) the fact that our analysis was based on grey matter tissue for which the PCASL is less sensitive to the ATT due to the small difference in T_1_ of blood and grey matter (Alsop and Detre, 1996). To avoid such a bias in the occipital lobe, one could obtain the individual baseline and hypercapnic ATT maps using a rapid low-resolution multi-PLD acquisition such as in Dai et al. (2012) to compute the true CBF value. Another solution would be to increase the PLD, while decreasing the number of slices such that the upper slices are not affected by a low SNR due to an excessively high ASL signal decay (De Vis et al., 2015, Wu et al., 2013). The use of simultaneous multi-slice acquisition may also help limit T_1_ decay of the ASL label signal over the volume.

## 5 Conclusion

Our project, in addition to examining pathophysiological changes in AD using calibrated fMRI, helped identify some key challenges related to calibrated fMRI such as the choice of the post-labeling-delay parameter to limit any flow-related bias due to delayed arterial transit time, the presence of susceptibility artifact, and the challenge associated with the hypercapnic manipulation in elderly participants, particularly those affected by dementia. Current challenges include: improving the characterization of vascular and metabolic burdens in the AD brain, along with providing additional information such as the transverse relaxation rate constant R2* and cerebrovascular reactivity to CO_2_. Future calibrated fMRI studies, in which the latter challenges are overcome, may provide new insights into the pathophysiology of AD that go beyond hyperphosphorylated tau pathology, amyloid deposition and glucose uptake. Moreover, calibrated fMRI may be of value in monitoring disease progression, in differentiating AD from vascular dementia, and for exploring mitochondrial dysfunction as a potential causal factor for the disease.
